# Using a soil bacterial species balance index to estimate potato crop productivity

**DOI:** 10.1371/journal.pone.0214089

**Published:** 2019-03-22

**Authors:** Thomas Jeanne, Serge-Étienne Parent, Richard Hogue

**Affiliations:** 1 Institut de recherche et de développement en agroenvironnement (IRDA), Quebec City, Quebec, Canada; 2 Université Laval, Quebec City, Quebec, Canada; Free University of Bozen-Bolzano, ITALY

## Abstract

The development of ‘molecular-omic’ tools and computing analysis platforms have greatly enhanced our ability to assess the impacts of agricultural practices and crop management protocols on soil microbial diversity. However, biotic factors are rarely factored into agricultural management models. Today it is possible to identify specific microbiomes and define biotic components that contribute to soil quality. We assessed the bacterial diversity of soils in 51 potato production plots. We describe a strategy for identifying a potato-crop-productivity bacterial species balance index based on amplicon sequence variants. We observed a significant impact of soil texture balances on potato yields; however, the Shannon and Chao1 richness indices and Pielou’s evenness index poorly correlated with these yields. Nonetheless, we were able to estimate the portion of the total bacterial microbiome related to potato yield using an integrated species balances index derived from the elements of the bacterial microbiome that positively or negatively correlate with residual potato yields. This innovative strategy based on a microbiome selection procedure greatly enhances our ability to interpret the impact of agricultural practices and cropping system management choices on microbial diversity and potato yield. This strategy provides an additional tool that will aid growers and the broader agricultural sector in their decision-making processes concerning the soil quality and crop productivity.

## Introduction

Soil microbial communities are impacted by meteorology [1,2], soil properties [3–5], agricultural management practices [6–9] and cropping systems [10,11]. In turn, the diverse composition of archaea, bacteria, fungi, protista and other eukaryotic communities found in soil and in the rhizosphere impacts the quality of soil, water and air resources [12], the degradability of organic matter [13] as well as the uptake of nutrients by plants [14,15]. Moreover, specific soil microbial groups can suppress soil-borne plant pathogens [16].

Doran and Parkin [17,18] defined *soil quality* as “the capacity of a soil to function within ecosystem and land-use boundaries to sustain biological productivity, maintain environmental quality, and promote plant and animal health”. According to Pankhurst et al., (1997) [19], the term *soil health* encompasses “the living and dynamic nature of soil, and captures the ecological attributes of the soil which have implications beyond its quality. These attributes are chiefly those associated with the soil biota: its biodiversity, its food web structure, its activity and the range of functions it performs”. Moebius-Clune (2016) [20] considered soil quality to include both inherent and dynamic soil properties and that soil health is equivalent to dynamic soil quality.

In agro-ecosystems, soil quality is assessed to enhance and sustain productivity. As for natural ecosystems, soil quality is monitored to favor the maintenance of environmental quality and biodiversity conservation [12]. A necessary requirement for soil quality assessment is the identification of sensitive soil attributes linked to soil functions [12]. To identify crop management effects, soil quality assessments must also include baselines.

Historically, soil quality is measured by monitoring abiotic, biochemical and biological indicators. Abiotic indicators include soil texture, wet aggregate stability, available water capacity, soil hardness, pH, cation exchange capacity and extractable nutrients. Biochemical indicators include organic matter, active carbon and the soil protein index. Finally, biological indicators include microbial respiration rate, nitrogen mineralization potential and microbial biomass [20–24]. These biological indicators provide no detailed information on soil biota at any taxonomic levels that may be impacted by the drivers and pressure factors identified in the Driver-Pressure-State-Impact-Response framework as applied to soil [12,25].

The lack of detailed information hinders the development of efficient land-use management strategies that could address the benefits humans derive from ecosystem services and the advantages and trade-offs derived from soil-based ecosystem services. The links between soil biota diversity/composition, soil functions and soil-based ecosystem services have been well established [12,26–29]. Microbial diversity and composition have been proposed as sources of biological indicators of soil health and agricultural soil quality [27].

Microbial communities play key roles in ecosystem processes by driving the Earth’s biogeochemical cycles and diversity metrics related to the richness, evenness, and phylogenetic diversity of soil microbial communities. They can be used to analyze the functional consequences of variations in soil microbial diversity [2,30]. Functional redundancy predicts that species loss does not necessarily alter ecosystem functioning since different species may exhibit overlapping functions [31]. The ecosystem functions performed by a number of microbial species may be less prone to the impacts of diversity loss [32]. Consequently, the functional redundancy impact may vary according to the ecosystem function(s) affected. For example, only minor changes in carbon mineralization were observed despite major shifts in the growth of soil bacteria and fungi [33]; yet, manipulations of soil microbial community composition produced heterogenous effects on ecosystem functioning, as measured by litter decomposition rates [34]. Terrat et al. [2] developed a predictive model of soil bacterial richness that incorporated bacterial taxonomic richness based on Operational Taxonomic Unit (OTU) numbers. These OTU numbers, determined by pyrosequencing 16S rRNA genes, were related to soil characteristics, climatic conditions, geomorphology, land use, and space variations across France. This French model provides a reference value of bacterial richness for a given pedoclimatic condition.

Among microbial taxonomic groups, some sharing similar functionality are influenced by agricultural practices. This suggests that, cropping practices may allow manipulation of influential community members [8]. Other investigators observed a shift in the flowering time of plant hosts that may coincide with the inoculation of early- or late-flowering soil microbiomes [35]. The reproducibility of the flowering phenotype across plant hosts suggests that microbiomes can be selected to modify plant traits and coordinate changes in soil resource pools.

The taxonomic composition of potato rhizosphere bacteria at various stages of plant development was stable in the face of very diverse environmental conditions [36]. An ‘opportunistic microbiome’ was identified which comprised OTUs that occur randomly or only under specific environmental conditions. In contrast, “core microbiome” OTUs were found at all sites. The ‘stable’ component of the core microbiome consisted of a few ubiquitous OTUs that were repeatedly abundant throughout all samples and vegetation stages, whereas the ‘dynamic’ component comprised OTUs that were enriched at specific vegetation stages [36]. The notion of OTU has recently evolved with the development of MiSeq targeted sequencing data preprocessing tools, such as DADA2 [37]. The new output unit defined has amplicon sequence variant (ASV) has improved our ability to define the basis of bacterial soil composition.

These recent studies spurred us to undertake this investigation, which has the following objectives: i) determine the physico-chemical, agronomic, climatic characteristics prevailing for each georeferenced soil sampled in 51 potato field plots; ii) determine the soil bacterial alpha- and beta-diversity of each soil sample; iii) apply an innovative selection procedure using physico-chemical, climatic and biological indicators to select amplicon sequence variants that correlate with potato yields; and iv) develop an integrative approach using a species balance index linked to potato productivity. The project’s paramount goal is to provide a state-of the-art tool that will assist agricultural stakeholders in their decision-making processes concerning the quality of soil and crop productivity.

## Materials and methods

### Soil sampling

This study was carried out on private agricultural lands. The owners of the agricultural lands are collaborators in our research project. They gave us permission to conduct this study on their lands.

During the 2013 and 2014 potato flowering period, 6 cm x 20 cm soil cores were collected from 51 geo-localized (GPS coordinates) sampling plots in the Province of Quebec in Canada. The plots were located in 13 potato fields. Each experimental plot was located at a corner of a 2500 m^2^ area in the field for a total of 4 samples per field. Each soil sample consisted of four soil cores (6 cm x 20 cm) taken on the row between two potato plants and each core was sampled at a corner of a 1m^2^ quadrant. The four soils cores of each sample were manually homogenized, and the soil samples were placed in sealed bags and kept on ice before being quickly stored at -80°C prior to DNA extraction. A summary of the samples is presented in Table 1.

**Table 1:** Summary of soil samples and soil properties (range of min-max values).

Field	Sample size	Potato class	pH	Total carbon	Total nitrogen	Soil Texture	Yield
	n			%	%		t/ha
F01	4	Russet(2)-Yellow(2)	4.56–4.75	1.59–2.38	0.13–0.17	Sand	18.75–23.11
F02	4	White round	4.95–5.38	2.59–3.32	0.18–0.27	Loamy sand—sandy loam	44.36–62.97
F03	4	Red round	5.30–5.40	1.56–2.38	0.15–0.27	Sand—loamy sand	26.73–35.86
F04	4	Russet	5.32–6.18	2.11–2.60	0.18–0.22	Sandy loam	33.35–53.48
F05	4	Russet	4.68–5.27	1.99–2.32	0.18–0.21	Sandy loam—loam	51.08–58.06
F06	4	White round	5.28–5.53	1.88–2.81	0.09–0.17	Sand—loamy sand	11.63–32.40
F07	4	White round	5.46–5.87	2.10–2.79	0.13–0.16	Loamy sand	30.34–41.97
F08	4	Red round	5.02–5.65	2.12–2.52	0.11–0.15	Loamy sand	23.43–38.29
F09	4	Red round	5.00–5.82	2.04–2.86	0.11–0.16	Sand—loamy sand	29.35–35.48
F10	4	Red round	4.98–5.70	1.79–2.85	0.10–0.18	Loamy sand	28.23–38.78
F11	4	White round	5.03–5.57	1.86–2.60	0.13–0.19	Loamy sand—Sandy loam	33.66–36.87
F12	3*	White round(2)-Yellow(1)	5.36–7.32	1.92–2.46	0.14–0.15	Loamy sand	22.70–35.26
F13	4	White round	5.04–5.70	0.99–2.45	0.04–0.11	Loamy sand—sandy loam	32.08–34.53

* One of the four soil samples of Field F12 was mishandled and unfortunately it has been discarded from the study.

### Soil characteristics

Soil physico-chemical analysis were performed on bulk soil of the 51 samples individually. Total C and N were measured after sieving dried soils at 100 mesh and were analyzed by combustion (Leco-CNS) [38]. Soil pH values were determined in water or in a 0.01M CaCl_2_ solution (1:1 v/v). Soil texture was determined by sedimentation [39]. The plots identified in the project are very representative of potato production in Quebec. We have a dominance of sand and loamy soils and a fairly variable range of potato yields.

### Soil bacterial composition

A 200 g aliquot of each soil sample was manually homogenized and sieved at 6 mm. Next, the 0.5 g sub-samples of 6 mm sieved soil were added to FastPrep-24 tubes containing 1.4 g of the beads matrix E and 1 ml of the lysis buffer supplied with the FastDNA SPIN Kit for Soil (MP Biomedicals, Solon, OH, USA). The DNA extraction step was performed according to the manufacturer’s instructions. Each DNA pellet was suspended in 100 μl of sterile molecular grade deionized water.

The quality and quantity of the DNA extracts were evaluated by spectrophotometry using a Biophotometer (Eppendorf, Mississauga, ON, Canada) with a G1.0 microcuvette μCuvette (Eppendorf, Mississauga, ON, Canada) with readings at 260, 280, 230, and 320 nm. Genomic DNA quality was also verified by electrophoresis on a 1.6% (w/w) agarose gel and visualized under UV with a Gel Doc XR+ instrument (Biorad, Hercules, CA., USA).

Prokaryota (archaea and bacteria) rRNA 16S (V4 region) gene was amplified using 515FB and 806RB primers [40–42] and a two-step dual-indexed PCR approach specifically designed for Illumina instruments by *Plateforme d’analyses génomiques* (IBIS, Université Laval, Quebec City, QC, Canada). Since bacteria are dominant, in this article text, the term bacteria will refer to bacteria and archaea. Briefly, the gene specific sequence was fused to the Illumina TruSeq sequencing primers and PCR was carried out in a total volume of 50 μl that contained 1X Q5 buffer (NEB, Whitby, ON, Canada), 0.25 μM of each primer, 200 μM of each dNTPs, 1 U of Q5 High-Fidelity DNA polymerase (NEB, Whitby, ON, Canada), and 1 μl of template DNA. The PCR began with an initial denaturation at 98°C for 30 s followed by 35 cycles of denaturation at 98°C for 10 s, annealing at 55°C for 10 s, and an extension at 72°C for 30 s with a final extension at 72°C for 2 min. The PCR reaction was purified using the Axygen PCR cleanup kit (Fisher Scientific, Nepean, ON, Canada). The quality of the purified PCR product was checked by electrophoresis on a 1% (w/w) agarose gel. Fifty- to one hundred-fold serial dilutions of this purified product were used as templates for a second PCR step with the goal of adding barcodes (dual-indexed) and missing sequences required for Illumina sequencing. The cycling parameters for the second PCR were identical to the first, but only 12 cycles were completed. PCR products were purified as above, checked for quality on a DNA7500 Bioanalyzer chip (Agilent, Santa Clara, CA, USA), and then quantified spectrophotometrically using the Biophotometer with a G1.0 microcuvette μCuvette. Barcoded amplicons were pooled in an equimolar concentration for sequencing on the Illumina MiSeq platform using a 2 X 300 bp sequencing kit.

After checking the quality of the run on the MiSeq instrument, the sequences obtained were demultiplexed according to the tag used. Next, sequence quality control and feature table construction were performed using QIIME 2 [43] and the dada2 plugin [37]. The SILVA 132 reference database [44] was used for taxonomic identification of amplicon sequence variants (ASVs). Here the Amplicon Sequence Variant (ASV) nomenclature replaces the well-known Operational Taxonomic Unit (OTU) appellation [45].

### Weather

Historical weather data were gathered from Environment Canada (http://climat.meteo.gc.ca) and transformed into weather indices, comparable to the procedure followed by Parent et al. [38]. The equations listed in Table 2 were used to compute the weather indices.

**Table 2:** Description of weather indices.

Index	Description	Unit	Formula
PPT	Cumulative precipitation	mm	$PPT\;=\;\sum_{i\;=\;1}^{n}{Rd}_{i}$
SDI	Shannon Diversity Index for rainfall	-	$SDI=\frac{-\sum_{i=1}^{n}\left[P_{i}\;ln\left(P_{i}\right)\right]}{ln\left(n\right)}$, $P_{i}=\frac{Rd_{i}}{PPT}$
GDD	Growing degree-days	°C	$GDD\;=\;\left\{\begin{array}{c} 0\;if\;{Tm}_{i}<7\\ \sum_{i\;=\;1}^{n}{Tm}_{i}\;if\;{Tm}_{i}\geq 7 \end{array}\right\}$

*Rd* is daily rainfall, *n* is the number of days and *Tm* is the daily mean temperature. The rainfall (SDI) equals 1 when daily precipitations are evenly distributed throughout the measured period and approaches 0 when precipitations occur on the same day.

### Statistics

#### Preprocessing soil compositions

Ever since John Aitchison provided solutions to spurious statistics using compositional data [46], the transformation from proportions to log-ratios has been widely adopted in soil [38,47,48] and life science studies [49,50]. The simplex constituted with proportions of carbon and nitrogen; as well as the non-overlapping mineral composition of sand, silt, and clay; was transformed into isometric log-ratios (*ilr*) [51] to generate balances of components. Balances were structured as strictly bifurcating trees [52] and computed with Eq 1:

$${ilr}_{k}\;=\;\sqrt{\frac{r_{k}s_{k}}{r_{k}{+s}_{k}}}ln\frac{{\left(x_{i_{1}}x_{i_{2}}\ldots x_{i_{r_{k}}}\right)}^{\frac{1}{r_{k}}}}{{\left(x_{j_{1}}x_{j_{2}}\ldots x_{j_{s_{k}}}\right)}^{\frac{1}{s_{k}}}},$$ (1)

where, for the *k*^*th*^ balance (*k* ∈ [1:*D*−1]) of a D-parts composition, *r*_*k*_ and *s*_*k*_ are the numbers of components in the respective right and left subsets of the *k*^*th*^ balance. Parts *x* with subscript *i* belong to the right subset in the numerator and parts *x* with subscript *j* belong to the left subset in the denominator. The coefficient $\sqrt{r_{k}s_{k}/\left(r_{k}+s_{k}\right)}$ is a normalization coefficient used to obtain an orthonormal domain.

**Fig 1:**
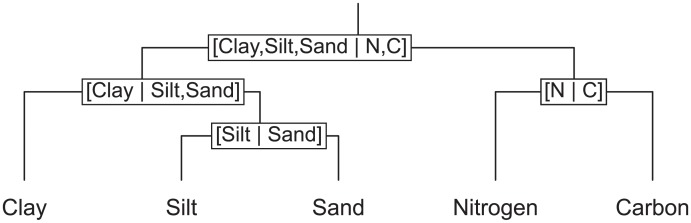
Balance structure used to compute soil isometric log-ratios.

As in Fig 1, balances are designated as [subset at denominator | subset at numerator], so that when the subset at the numerator is larger than the subset at the denominator, the *ilr* value is positive and conventionally placed to the right of the axis.

#### Preprocessing bacterial communities

Recent research [53–55] stresses the importance of preprocessing microbial community data with a compositional transformation, which requires imputing zeros with pseudo-counts [56] namely using Bayesian-multiplicative replacements [57]. A feature-table was filtered by retaining only ASVs with a contingency of 2 to reduce the number of zeros in the table prior to transforming the data into centered log-ratios (*clr*) ASVs done with the feature-table plugin implemented in QIIME 2.

We transformed ASV counts to *clrs* [46] using Eq 2:

clrk=log(xkx1×x2×…×xk1k) (2)

#### Statistical analysis workflow

**Fig 2:**
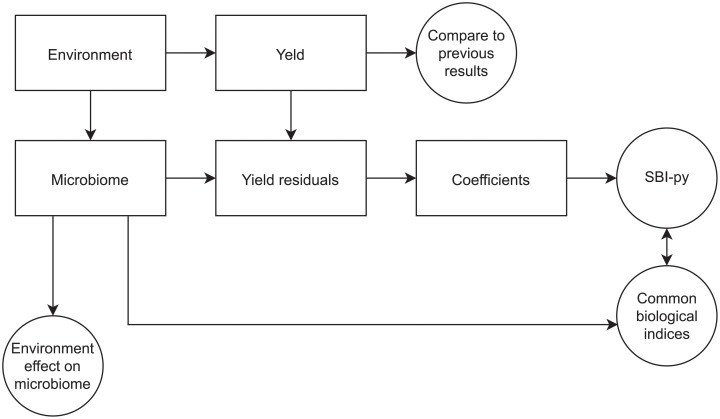
Statistical analysis workflow applied to soil microbiome and potato crop productivity.

We designated potato yield as a performance index. Environmental variables, i.e. weather and soil, are likely to affect yield, so a linear regression was performed (i) to compare the effects of environmental variables with the effects obtained in a similar study previously carried out in the Province of Quebec, Canada [38], and (ii) to obtain a residual yield, i.e. the part of the yield unexplained by environmental variables. Then a correlation analysis was run between the *clr*-transformed bacterial microbiome and the residual yield. In order to calculate a bacterial species balance index of potato productivity (SBI-py), we computed a log-ratio of ASVs whose *clrs* were positively (numerator) and negatively (denominator) correlated to residuals yield, at a significant 0.05 level. This index was compared to the Shannon and Chao1 biological diversity indices as well as to Pielou’s evenness index. These diversity indices are commonly used to assess alpha diversity [58–60]. Finally, we attempted to detect ecological niches related to SBI-py by analyzing the effect of the environmental variables on potato productivity. The analysis workflow is presented in Fig 2.

#### Computational environment

Statistical computations were performed in the R version 3.5.0 [61]. The tidyverse version 1.2.1 meta-package was used for generic data analysis. The vegan package version 2.5–1 [62] was used to compute the redundancy analysis. The compositions package version 1.4.1 [63] was used for soil isometric log-ratio transformations. The zCompositions package version 1.1.1 [64] was used to impute counted zeros in the ASV table. The data and the R code are both available at https://git.io/fhHEj.

## Results and discussion

The 51 samples of the 13 fields described in Table 1 were distributed between sand to loam textures (Sand: 30; Loamy sand: 8; Sandy loam to Loam: 13) and 4 classes of potato (Red round:18; White round: 20; Russet: 11; Yellow:3). The quality of the data is very good and meets the quality criteria to allow a microbial diversity analysis. After the application of the DADA2 pipeline, the average sequence rate per sample was 18613 for a total of 949242 sequences for the 51 samples. 2008 different ASVs were observed in the dataset.

### Regressions analysis of potato yield with meteorological and physico-chemical variables

**Fig 3:**
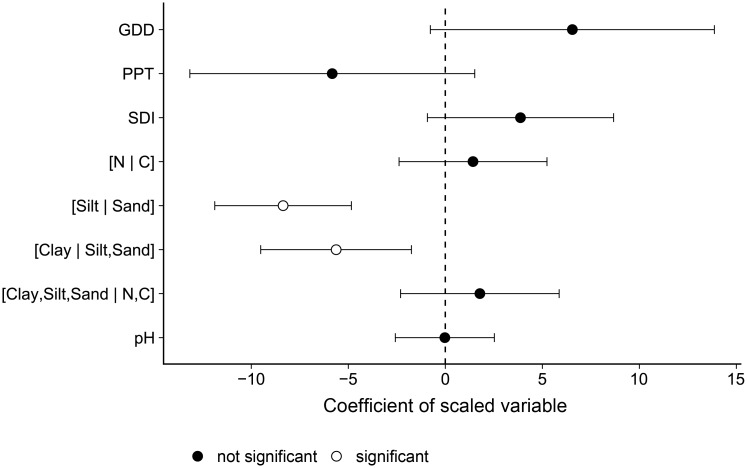
Slope coefficients and their 95% confidence intervals from a linear model linking environmental and physico-chemical features to potato yield.

A regression model was used to analyze the relationship between the selected weather and chemical variables and the potato yield. The slope coefficients of the scaled and centered variables are shown in Fig 3.

The two textural balances, [Clay | Silt, Sand] and [Silt | Sand] displayed significant negative slope coefficients at the 0.05 level. This implies that the increase in sand content in the soil relative to clay (i.e., the [Clay | Silt, Sand] balance) is negatively correlated to potato yield, or in other words, the relative proportion of clay compared to non-cohesive particles is positively linked to yield. Also, a higher proportion of sand compared to silt (i.e., the [Silt | Sand] balance) is negatively correlated to yield. Otherwise, no significant coefficients with variables linked to the [N | C] balance, pH, and weather were observed.

Once the residuals of the regression, here referred to as the *residual yield*, are extracted they can be related to the biological components of the soils.

### Correlation between soil bacterial composition and residual potato yield

We correlated the *clrs* of ASV counts with residual potato yields. From these correlations, we retained ASVs associated to significant correlations at the 0.05 level. We identified 79 positively correlated and 61 negatively correlated ASVs from a total of 2008.

**Fig 4:**
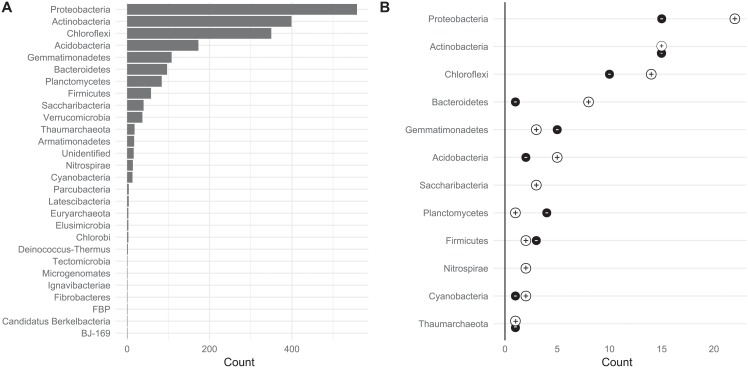
(A) Counts of ASVs per bacterial phyla (bacteria and archaea) detected in soil samples and (B) counts of ASVs per bacterial phyla that significantly correlated, either positively or negatively, with residual yields at the 0.05 level of significance.

The Fig 4A shows the counts of ASVs identified per bacterial phyla from the bacterial composition of soil samples, while Fig 4B shows the number of positively and negatively correlated ASV in non-neutral phyla.

Retained ASVs belong to 12 bacterial phyla. The total and retained abundance patterns of ASV were similar, although *Verrucomicrobia*, *Armatimonadetes* and other less abundant phyla were absent from the retained ASVs. The most of retained ASVs were from the *Proteobacteria*, *Actinobacteria*, *Chloroflexi*, *Bacteroidetes*, *Gemmatimonadetes* and Acidobacteria phyla. In the case of *Proteobacteria*, *Bacteroidetes* and *Acidobacteria*, there was a higher number of retained ASVs that positively correlated with residual potato yield. Among the the *Saccharibacteria and* Nitrospirae phyla, we observed no *clrs* of ASVs counts that negatively correlated with the residual potato yield. This suggests that among these six bacterial phyla, most retained ASVs contribute positively to potato crop productivity.

### Relationship between potato yield and soil bacterial diversity indicators

**Fig 5:**
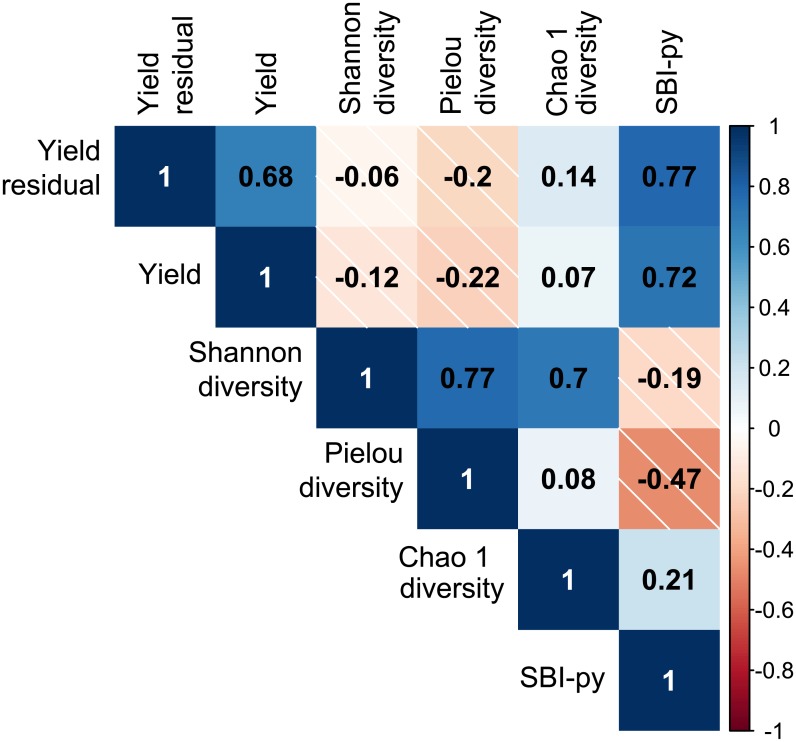
Relationships between alpha diversity metrics and the *SBI-py* index with residual potato yield.

A *species balance index related to potato yield (SBI-py)* was computed as the log-ratio between the ASV counts associated to positive (numerator) and negative (denominator) significant correlations (at the 0.05 level) between ASV *clrs* and potato residual yields. Fig 5 illustrates the correlations between potato yield, residual potato yield and three commonly used alpha diversity indices (Shannon diversity, Pielou’s evenness and Chao 1 diversity), as well as the SBI-py.

The three alpha diversity indices were barely correlated with potato yield and residual potato yield. The Pielou index correlated less well with potato productivity than did the other two alpha diversity indices. Alpha diversity indicators have been used to assess impact of land uses [2] and of diverse soil management practices and cropping systems on soil quality [16,27,65]. The comparison of organic farming vs conventional agriculture [8,66] and of different farming systems [10,67] showed significant differences of alpha diversity indices values. In our study, the relative homogeneity of the cropping systems and management practices may explain that no links between bacterial alpha diversity indices and the potato residual yield have been observed.

It has been reported that specific guilds of taxa among the soil bacterial microbiome can be selected to modify plant traits and to coordinate changes in soil resource pools [7,35,36]. These reports spurred us to identify specific bacterial species/ASVs among soil bacterial composition linked to potato productivity. Since the influence of soil properties and weather conditions on the microbiome is widely known [68,69], we first performed a linear regression with potato yield to get a residual yield that was not affected by the weather or soil parameters. Then the *clrs* of ASV counts that correlated positively or negatively with this residual yield have been used to determine a *species balance index related to potato yield (SBI-py)*. This innovative selection procedure of ASVs lead by design to good correlations between the newly developed species balance index (SBI-py) and the residual potato yield of the field plots sampled.

Burns et al., [7] reported a relative hierarchy of effect of vineyards management practices, cropping systems, soil properties and soil resource pools on microbial community structure based on NMDS analysis using coarse and fine (genus) taxonomic level.

Also, a detailed statistical analysis on the operational taxonomic unit (OTU) level, representing bacterial species detected in potato rhizosphere, revealed a stable component and a dynamic component of a core microbiome related to potato crop and field sites sampled [36]. As observed in our study based on bulk soil prokaryotic microbiome analysis, the diversity metrics of potato rhizosphere bacterial community showed that the β-diversity was more significantly modified than the α-diversity [36].

The use of ASV instead of OTU tables or taxonomic tables allowed a better appreciation of the complexity and species variability among soil microbiomes. The accuracy of the taxonomic tables (phylum, class, order, family or genus levels) is limited due to the reference databases that are used for each OTU or ASV taxonomic assignations. This limitation is even more important with the use of OTUs made with 97% similarity between sequences instead of ASVs.

### Effect of environmental variables on the SBI-py

**Fig 6:**
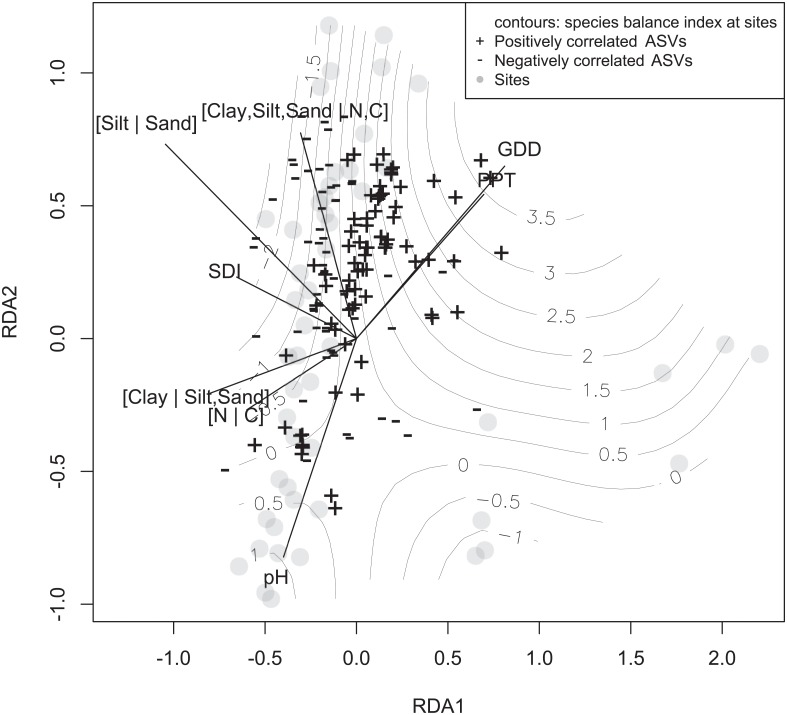
Triplot of a redundancy analysis showing ecological niches of ASVs that exhibit a significant positive (+) or negative (-) correlation with potato yield residual, as well as those ASVs with no significant correlation (translucid points) in distance scaling (scaling 1).

The triplot shown in Fig 6 results from a redundancy analysis between environmental variables, soil physico-chemical balances and the *clrs* of ASVs. The positively and negatively significantly (at the 0.05 level) correlated *clr*-transformed ASV counts at each site (greyish dots) are plotted along the contours of the species balance index scores. By design, the SBI-py contours follow the pattern of positive and negative ASVs.

The obvious delineation of positive and negative ASVs zones indicate potential ecological niches related to favorable and unfavorable soil microbiomes for potato cropping systems. Because the [Clay | Silt, Sand] balance vector points in an opposite direction of the high SBI-py positive values, we infer that soils with higher clay proportions are linked to higher SBI-py positive values. The same reasoning applies to the [N | C] balance: higher nitrogen to carbon content is linked to higher SBI-py positive values. On the other hand, high organic contents, ([Clay, Silt, Sand | N, C] balances) and sandy soils (high [Silt | Sand] balances) are linked to negative SBI-py index values. The Shannon diversity index of rainfalls [SDI] points through a negative SBI-py values, indicating that a more even distribution of precipitations is linked to low SBI-py values. Finally, although the pH vector does not follow the highest SBI-py gradient, we can find relatively high SBI-py values at low pH.

## Conclusions

Our results highlight the importance of using soil bacterial composition as a biological index of soil quality and, more specifically, of crop productivity. We developed the SBI-py index based on the evidence that specific components of the soil microbiome can explain aspects of potato productivity. While, by design, the SBI-py showed a high correlation with yield detrended from several environmental conditions (0.77), three commonly used alpha-diversity indices (Shannon, Chao1 and Pielou’s evenness indices) poorly correlate to potato productivity. The approach we employed to develop the SBI-py index could be further validated using a larger sample size combined with machine learning techniques.

The soil microbiome encompasses different prokaryotic and eukaryotic organisms dispersed along a complex interacting food web, thus providing numerous soil ecosystemic functions that may promote plant growth [70]. For example, the potato tuber yield was impacted by the fungal diversity in rhizosphere soil of continuous cropping potato subjected to different furrow-ridge mulching managements (71). The rhizosphere soil under the on-ridge planting with full-mulch (T2) soil had the highest fungal diversity and the highest potato yield.

We have recently undertaken a study of the interactions between bacterial and fungal diversity in soil and rhizosphere of potato cropping systems to evaluate their impact on potato yield and soil productivity.

Our results represent a significant contribution to research aimed at selecting biotic data that give more detailed information on soil biota than the usual biological indicators. These biotic data can be incorporated as biological indicators into the determination of soil quality indices. Our strategy for the selection of significant ASVs in specific component of soil microbiome can also be implemented in a variety of agricultural applications.
